# In-feed Praziquantel treatment of grass carp (*Ctenopharyngodon idella*) infected with eye flukes

**DOI:** 10.17221/97/2023-VETMED

**Published:** 2024-01-23

**Authors:** Eliska Zuskova, Josef Velisek

**Affiliations:** South Bohemian Research Centre of Aquaculture and Biodiversity of Hydrocenoses, Faculty of Fisheries and Protection of Waters, University of South Bohemia in České Budějovice, Vodňany, Czech Republic

**Keywords:** anthelmintic, fish, diplostomosis, *Diplostomum pseudospathaceum*, medicated feed

## Abstract

Praziquantel (PZQ) is a drug effective against several parasitic diseases of fish caused by Platyhelminthes. The study assesses the efficacy of selected in-feed PZQ doses in eliminating metacercariae *Diplostomum pseudospathaceum* in grass carp. The fish were infected with *D. pseudospathaceum* in laboratory conditions and subsequently treated with PZQ at daily doses of 30 (PZQ1), 50 (PZQ2), and 100 (PZQ3) mg/kg bw for seven days. The treatment trial was followed by a 23-day observation period during which the fish were parasitologically examined on days 0, 2, 8 and 23. Smaller fish were infected with a significantly higher (*P* < 0.05) number of metacercariae than bigger fish among three weight groups as follows: up to 7 g > 7 to 12 g > 12 g. The in-feed PZQ at doses of 30, 50 and 100 mg/kg bw for seven days was 47%, 84% and 88% effective against metacercariae *D. pseudospathaceum*, respectively. A significant decrease (*P* < 0.01) in moving and an increase (*P* < 0.01) in the immotile metacercariae in the eye’s lens were recorded in all the trial groups on each sampling day. An increasing proportion of motile metacercariae in the post-treatment observation period was seen in PZQ2 and PZQ3, indicating a decreasing trend in the actual efficacy in an environment with no reinfection possibility.

Trematodes of the genus *Diplostomum* (von Nordmann 1832) belong to the most common parasitic infections in pond-stocked fish, causing considerable losses, particularly in younger fish species of littoral habitats (offshore zones with plants). The life cycle of *Diplostomum* sp. Includes passage through birds, snails and fish (Chappel et al. 1994). Free-living stages (cercariae) are released in high numbers from snails and infect the lens of the fish eye, where they are transformed into the larval stage called metacercariae (Karvonen 2012). While in the lens, metacercariae cause cataracts (Shariff et al. 1980; Karvonen et al. 2004), impairing the vision of the fish and leading to a reduced feeding ability and growth (Crowden and Broom 1980; Owen et al. 1993; Buchmann and Uldal 1994). The tissue migration from penetration to establishment in the lens is typically completed within 24 h (Whyte et al. 1991), but can take longer in low temperatures (Lyholt and Buchmann 1996). In one fish, tens to hundreds of metacercariae can parasitise in the eyes, where infections accumulate over time (Marcogliese et al. 2001). The problem of reduced visual orientation and, consequently, reduced food intake has been noted, especially in the wild, as well as in farmed salmonid fish, which preferentially use vision to find food (Marcogliese et al. 2001; Seppanen et al. 2008; Seppala et al. 2011). A different situation occurs with infected cyprinid fish from a pond environment. Cyprinids are not limited by their reduced visual function since they are artificially fed and do not have to look for food; however, there is a change in the behaviour of the attacked host (colour change, movement near the surface), which can then become the preferred prey of a potential definitive host, a fish-eating bird. Recently, the biggest problems with diplostomosis due to secondary predation (up to 100%) have been reported in young categories of grass carp (*Ctenopharyngodon idella*), which could be easily infected in the littoral zones of ponds.

Praziquantel (PZQ) is a broad-spectrum pyrazino-isoquinoline anthelmintic effective against many platyhelminth parasites in the aquaculture industry (Norbury et al. 2022). Since PZQ is not listed for use in fish for human consumption in the EU, its use in aquaculture should be “off-label” under special veterinary justification within the maximum residue limits, withdrawal periods and safety estimation in the target fish species. Its safety (Zuskova et al. 2022) and efficacy against *Diplostomum* spp. Has been confirmed in several studies (Bylund and Sumari 1981; Plumb and Rogers 1990; Szekeli and Molnar 1991; Zuskova et al. 2018), where PZQ was applied both orally and as a bath in various fish species. Due to the difficulty of performing therapeutic baths (Kolarova et al. 2022) and the effort to minimise the burden on the environment, feeding with medicated feed appears to be the most suitable and effective way to eliminate the infection. The study aimed to verify the efficacy of the in-feed administered antiparasitic praziquantel on the controlled infection of *Diplostomum* spp. in grass carp.

## MATERIAL AND METHODS

### Experimental animals

Three hundred parasite-free grass carp individual (*Ctenopharyngodon idella*) hatchlings were reared from eggs to experimental size (mean weight = 6.7 ± 2.4 g) at the Research Institute of Fish Culture and Hydrobiology (RIFCH) hatchery in Vodňany, Czech Republic. The commercial diet Premium select (Alltech Coppens, the Netherlands) was used as the control and basal diet for all the treatment diets.

### Infected intermediate hosts

Cercariae of *Diplostomum pseudospathaceum* were obtained locally from naturally infected snails *Stagnicola* spp. Collected from the experimental fish ponds in Vodňany, Czech Republic.

Immediately after collection, the snails were stimulated with light to produce cercariae with a subsequent species identification assessment based on the morphological features described by Selbach et al. (2015). The infected snails were kept in the laboratory in a 25 l glass aquarium under a 12 : 12 h light : dark cycle at 18 °C in aerated tap water and were fed lettuce *ad libitum* in accordance with Haas et al. (2002). Cercariae were shed from *Stagnicola* spp. By bright light exposure for 2 h (Whyte et al. 1990) and the density of the cercarial suspension was estimated volumetrically.

### Preparation of medicated feed

The praziquantel (PZQ) powder from Ecological Laboratories Inc. (Cape Coral, USA) was used to prepare the medicated feed. The commercial control diet was crushed, supplemented with 5 ml of fish oil to mask the “flavour” of the anthelmintics and mixed with PZQ (1.5 g, 2.5 g and 5 g per kg of feed) before the pelleting procedure in a common feed pelletiser and cold extruder according to the methodology of Roy and Mraz (2021). Subsequently, the formed and dried pellets were sprayed with gelatin, protecting the pellets from rapid disintegration in the water. The same process was used for the control with the exclusion of PZQ. Each dose of PZQ for each aquarium was calculated from the mean weight of the fish. The PZQ concentrations were confirmed by liquid chromatography-mass spectrometry (LC-MS/MS) using the method of Baralla et al. (2020).

### Experimental set-up

#### FISH INFECTION

Three hundred fish were placed into the aquarium with a volume of 40 litres of dechlorinated tap water for direct infection. A volume of 400 ml of a *D.* *pseudospathaceum* cercarial suspension (mean density of 20 cercariae in 1 ml) was added to the fish and left for 2 h at 18 °C without direct light. Because the stage in the lifecycle of the flukes highly affects their infectivity (Karvonen et al. 2003), every care was taken to ensure that the cercariae introduced into aquarium were alive, active, and at the same age (3 h after being released from the snails) at the beginning of the infection trial. The dispersion of cercariae in the aquarium was ensured by continuous aeration. Subsequently, 300 ml of water was added the following three days. The infection success was quantified in twelve fish by counting the number of *D.* *pseudospathaceum* metacercariae immediately before the treatment trial.

#### TREATMENT TRIAL

Two hundred eighty-eight fish were weighed and equally divided between twelve 200 l glass aquaria (*n* = 24 fish per triplicate tank) filled with dechlorinated tap water in a randomised block design.

The water temperature, oxygen saturation and pH were measured daily in each aquarium and ran-ged from 16.0 to 17.5 °C, 87–95%, and 7.1–7.8, respectively. The mean daily ration consumed over 14 days prior to the infection was used as the ration for the trial. The three concentrations of PZQ medicated diet (1.5 g, 2.5 g, and 5 g per kg of feed) and an untreated control were fed at 2% body weight (b.w.) (calculated for each aquarium) once a day for a 7-day study duration to achieve the treatment doses of:

1. PZQ1: 30 mg/kg bw per day.
2. PZQ2: 50 mg/kg bw per day.
3. PZQ3: 100 mg/kg bw per day.
4. C: control diet.

The treatment trial was followed by a 23-day observation period during which the fish were parasitologically examined on days 0, 2, 8 and 23 after the last medicated feed administration. For the parasitological examination, 18 fish were taken from each group (6 from each aquarium).

### Parasitological examination

The fish were killed, and the eye lenses were removed and carefully pressed between slides. The number of moving and immotile metacercariae were counted under a light microscope equipped with a camera, type E-600 (Olympus BX51, Tokyo, Japan). Motility was assessed by observing every parasite individually for 30 s under light microscopy and a single observer recorded the presence/absence of motility. The prevalence (%) and abundance of moving (MA) and immotile (IA) metacercariae (the average number of parasites found per each examined fish from the group) of the *Diplostomum pseudospathaceum* infection were evaluated.

The fish sampling and protocols of this study were conducted in compliance with Czech regulations 166/1996 and 246/1992 and approved by the Departmental Expert Committee for Authorisation of Experimental Projects of the Ministry of Education, Youth, and Sports of the Czech Republic (Permit MSMT-3126/2021-3).

### Statistical analysis

The statistical software program STATISTICA (v8.1 for Windows, StatSoft, USA) was used to compare the differences among the test groups. The efficacy of each treatment was assessed as a percentage reduction in the mean abundance relative to the control groups and was calculated by adapting the formula of Stone et al. (1999):

$$\%\text{ reduction }(\text{efficacy})=100-(100\times \frac{\begin{array}{c} \text{mean moving parasite}\;\\ \text{abundance of the}\;\text{treated}\;\\ \text{group} \end{array}}{\begin{array}{c} \text{mean moving parasite}\;\\ \text{abundance of the control}\;\\ \text{group} \end{array}}$$ (1)

The mean abundances were generated for each replicate. The differences in the number of metacercariae and the weight of the fish in the control were analysed. Prior to the analysis, all the measured variables were evaluated for normality (Kolmogorov-Smirnov test) and for the homoscedasticity of variance (Bartlett’s test). If these conditions were satisfied, a one-way analysis of variance (ANOVA) was employed to determine whether there were significant differences in the measured variables among the experimental groups. When a difference was detected (*P* < 0.05), Dunnett’s multiple range test was applied. If the conditions for the ANOVA were not satisfied, a non-parametric test (Kruskal-Wallis) was used.

## RESULTS

The fish ingested the medicated feed successfully without any avoidance behaviour, even at the highest dose. There was no mortality and no behaviour changes during the trial.

**Figure 1:**
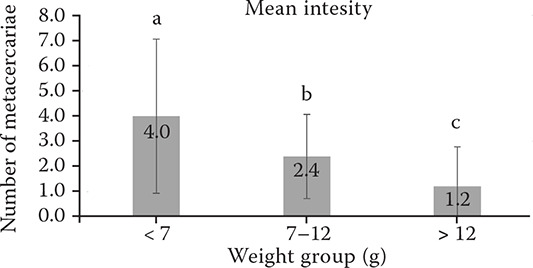
The mean intensity of the infection in the control fish divided into three groups according to weight (< 7 g, 7–12 g, and > 12 g) Different letters represent significant differences (*P* < 0.05) between the groups

The infection success rate (prevalence) in the pre-trial group (*n* = 12) was 83%, with an average abundance of 3.2 metacercariae per fish. In the trial control groups, the prevalence ranged from 78–89%, with an average abundance of 2.9 metacercariae per fish. The intensity of the infection in all the control fish, divided according to the weight into three groups (< 7 g, 7–12 g, and > 12 g), significantly decreased (*P* < 0.05) in the heavier groups (Figure 1).

The prevalence in the trial groups exceeded 60%. The values of the parasite prevalence and abundance of the infection calculated for the moving and immotile metacercariae are shown in Table 1.

**Table 1:** Prevalence and the mean abundance of the moving (MA) and immotile (IA) metacercariae *D. pseudospathaceum* in the fish lens of the control I and trial groups (PZQ1, PZQ2 and PZQ3) sampled on days 0, 2, 8 and 23 after treatment

Time	Parameter	C (*n* = 18)	PZQ1 (*n* = 18)	PZQ2 (*n* = 18)	PZQ3 (*n* = 18)
0	Prevalence (%)	78	89	67	67
	Abundance-MA (x ± SD)	2.2 ± 1.6	1.5 ± 1.8	0.2 ± 0.4	0.1 ± 0.2
	Abundance-IA (x ± SD)	0	1.2 ± 1.7	1.5 ± 1.7	2.3 ± 3.0
2	Prevalence (%)	83	67	78	67
	Abundance-MA (x ± SD)	4.1 ± 3.6	1.3 ± 2.0	0.1 ± 0.5	0.2 ± 0.4
	Abundance-IA (x ± SD)	0	0.8 ± 1.5	1.7 ± 2.0	1.5 ± 2.0
8	Prevalence (%)	83	83	78	67
	Abundance-MA (x ± SD)	2.2 ± 1.5	1.3 ± 1.5	0.4 ± 0.5	0.3 ± 0.7
	Abundance-IA (x ± SD)	0	0.6 ± 0.9	1.6 ± 1.0	2.2 ± 2.2
23	Prevalence (%)	89	94	61	72
	Abundance-MA (x ± SD)	3.0 ± 2.3	1.8 ± 1.3	1.1 ± 1.2	0.7 ± 0.8
	Abundance-IA (x ± SD)	0	2.5 ± 4.3	1.4 ± 2.4	1.3 ± 2.3

**Figure 2:**
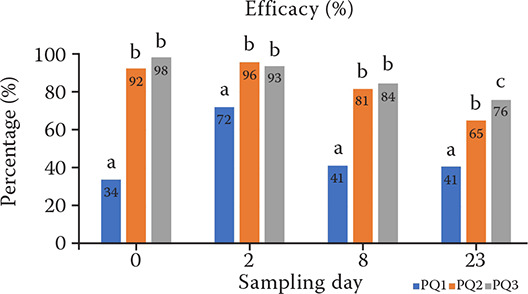
The efficacy of the PZQ treatment in the doses of 30 (PZQ1), 50 (PZQ2) and 100 (PZQ3) mg/kg of body weight for 7 days mentioned on the post-treatment sampling days 0, 2, 8 and 23 Different letters represent significant differences (*P* < 0.01) between the groups for each sampling day (0, 2, 8 and 23)

The mean efficacy calculated from all the sampling days was 47%, 84% and 88% for the PZQ1, PZQ2 and PZQ3 groups, respectively. The efficacy of the PZQ treatment was significantly higher (*P* < 0.05) in the PZQ2 and PZQ3 groups compared to PZQ1 on sampling days 0, 2 and 8. A statistically significant difference (*P* < 0.01) in efficacy was recorded between all the trial groups on the last sampling day 23 (Figure 2).

**Figure 3:**
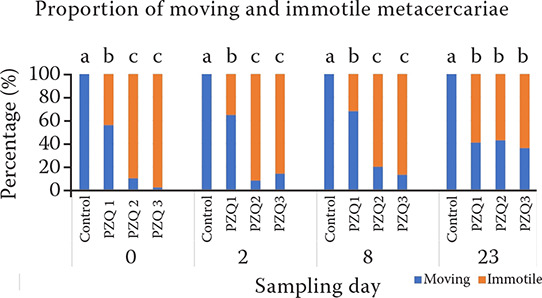
The proportion of moving and immotile metacercariae in the control and trial groups PZQ1, PZQ2 and PZQ3 on each sampling day Different letters represent significant differences (*P* < 0.01) in the numbers of moving and immotile metacercariae for each sampling day (0, 2, 8 and 23)

A significant decrease (*P* < 0.01) in the moving metacercariae and a significant increase (*P* < 0.01) in the immotile metacercariae was mentioned in the trial groups PZQ1, PZQ2 and PZQ3 compared to the control on each sampling day. The proportion of moving and immotile metacercariae is shown in Figure 3. The post-treatment trend of an increasing portion of moving metacercariae over time was the most evident in the PZQ2 and PZQ3 groups. The percentage of moving metacercariae was stable in the PZQ1 group with the overall sampling.

## DISCUSSION

Diplostomosis of fish is a problematic infection to eradicate since it has an indirect complex life cycle and its controlled medication is inappropriate in open systems. The oral administration of PZQ mixed in feed can be the most convenient and cost-effective form of delivery, especially when treating a large fish population (Norbury et al. 2022). The main disadvantages of oral administration include the unequal distribution of the dose in the fish population, where parasitised animals may have a reduced appetite (Parker et al. 2023), individual competition for food present (Chuard et al. 2018), or a feed may be unpalatable (Al-Souti et al. 2019). Despite the bitter taste and low palatability of PZQ, we did not notice any avoidance behaviour in the fish, even at the highest PZQ dose.

Diplostomosis is particularly dangerous in smaller fish, which are more easily infected when cercariae pass through the skin or gills. According to Kuukka-Anttila et al. (2020), rainbow trout (*Oncorhynchus mykiss*) with lower resistance to parasites (with higher metacercaria infection rates) had a lower body weight. This finding was confirmed by the lower average abundance of metacercariae in the heavier groups of fish in our study. On the other hand, Unger et al. (2022) proved that rainbow trout from open farm systems accumulate eye flukes over time and independent of the differences in resistance, getting exposed to a constant infection rate with the cercariae throughout the year. PZQ is effective at treating the juvenile stages of digeneans in fish, with the treatment used to avoid detrimental health impacts, to maintain the market value, or to disrupt the parasite life cycle (Bader et al. 2019). In-feed PZQ at doses 30, 50 and 100 mg/kg of body weight for seven days was 47%, 84% and 88% effective against the *D. pseudospathaceum* infection, respectively. A 100% efficacy was achieved by Szekely and Molnar (1991) in their study on grass carp, where a single dose of 330 mg/kg bw was orally applied. However, lower doses of PZQ should minimise any palatability issues (Bader et al. 2019) and a repeated administration ensures a greater possibility of distribution within the population under field conditions.

The increasing number of moving metacercariae after treatment suggests only the temporary paralysis of some parasites. The actual efficacy of the PZQ administration cannot be objectively assessed immediately after application, but rather after a few weeks. The mechanism and dynamics of removing dead parasites from the eye lens are still unclear.

Considering the life cycle of *D. pseudospathaceum* in pond ecosystems, cercariae continuously infect the resident fish. At a high infection intensity, there is a change in the behaviour of the attacked host (colour change, movement near the surface), which can then become the preferred prey of a potential definitive host, a fish-eating bird, e.g., a seagull. The escape velocity is also affected compared to healthy fish (Seppala et al. 2004; Rintamaki-Kinnunen et al. 2004). This phenomenon, where a parasite changes the behaviour of its host to increase the probability of its transmission to another host, is called Parasite-Increased Trophic Transmission (PITT). This phenomenon mainly affects grass carp under one year, where massive mortality of infected fish due to the highest predation pressure is present. It is advisable to treat this most endangered category repeatedly during the summer period when there is the most significant release of infectious developmental stages (cercariae) from intermediate-host snails (Haas et al. 2002). Therefore, the treatment strategy for small grass carp should not be completely eliminating the parasitic infection from fish kept in an infested environment, but the therapeutic bridging of the riskiest breeding phase. The seven times repeated PZQ dose of 50 mg/kg b.w. appears to be the most appropriate for the practical limitation of *D. pseudospathaceum* infection in younger age grass carp, where the feed palatability is minimally affected and a sufficient high treatment efficiency (65%) remains even after 23 days.
